# Modeling organizational intelligence, learning, forgetting and agility using structural equation model approaches in Shiraz University of Medical Sciences Hospitals

**DOI:** 10.1186/s13104-021-05682-w

**Published:** 2021-07-21

**Authors:** Zahra Kavosi, Sajad Delavari, Mohammad Mehdi Kiani, Peivand Bastani, Mohebat Vali, Marzieh Salehi

**Affiliations:** 1 Health Human Resources Research Center, School of Management & Information Sciences, Shiraz University of Medical Sciences, Shiraz, Iran; 2 Department of Health Management, Policy & Economics, School of Public Health & Health Equity Research Centre (HERC), Tehran University of Medical Sciences, Tehran, Iran; 3 Student Research Committee, Shiraz University of Medical Sciences, Shiraz, Iran

**Keywords:** Hospital, Organizational agility, Organizational forgetting, Organizational intelligence, Organizational learning, Structural equations

## Abstract

**Objectives:**

Strengthening the organizational agility of the hospital can lead to decreased production costs and increased market share, better serving to patients' needs, introduction of new services and increased competitiveness; therefore, this study aimed to investigate the agility of Shiraz public teaching hospitals.

**Results:**

The results showed that organizational intelligence had a positive and significant effect on organizational agility with a path coefficient of 0.172. Organizational forgetting and organizational learning also played a mediating role between organizational intelligence and organizational agility. This means that organizational intelligence had positive effect on organizational forgetting with path coefficient of 0.482, organizational forgetting on organizational learning with path coefficient of 0.40 and subsequently organizational learning on organizational agility with path factor of 0.07. Organizational forgetting also played a mediating role between organizational intelligence and organizational learning.

**Supplementary Information:**

The online version contains supplementary material available at 10.1186/s13104-021-05682-w.

## Introduction

In today's bubbly and Variable world, the only thing that doesn't change is change. Organizations are increasingly under-effected by the three words customer, competition and change, and are always looking for ways to overcome these problems [1]. If we look at the hospital as an organization, then we can conclude that due to the competitive environment around the customers can choose other service centers and for the sake of survival and sustainability, the organization has to change and adapt to existing conditions, adopt a competitive environment and take advantage of economic mechanisms [2].

Organizations must seek an organizational agility paradigm to maintain their competitive ability and to cope with change. In government organizations, agility is very important because the results and successes of these types of organizations are interesting and admirable. The results of a study conducted by Jafarinejad and Shahaei [3] entitled of agility capabilities in government organizations showed that agile government institutions has grown and increased in productivity 53%, in staff satisfaction 38% and in customer or consumer satisfaction 3% [3].

The term organizational agility was first coined in 1991. Agility means the ability to respond quickly and successfully to environmental change [4]. Existing studies and experience show that strengthening the organizational agility of the hospital can lead to increased production costs and increased market share, better serving to patients' needs, introduction of new services, and increased competitiveness. Therefore, agile hospital has become a popular concept in health systems and competitive markets of hospital services. Therefore, current health managers try to improve the agility of their organizations more than ever before [5]. Grol et al. [5] argue that health organizations need to use different frameworks that combine strategies, activities, and actions for agility [6]. Organizational agility is further identified by four components: (1)response, (2) Competency, (3) Flexibility, (4) Speed.

Studies have identified several factors affecting organizational agility. According to these studies, variables of organizational learning, organizational intelligence and organizational forgetting had the highest causality relationship with organizational agility variable [7, 8].

By examining the components of organizational intelligence can identify the status of the organization in terms of level of intelligence i.e. compatibility and adapt to the environment, perspectives, learning and application of knowledge, organizational structure and performance, moral, information and communication technology and organizational memory. And then, by focusing on capabilities and planning to address weaknesses, improve the efficiency and effectiveness of the organization. From al-Barakht's perspective, organizational intelligence can be identified with 7 components [9]. (1) Strategic approach, (2) Common file, (3) Tend to change (4) Heart, (5) Alliance, (6) Spreading of knowledge. (7) Pressureof performance based on research conducted organizational intelligence can have a direct impact on organizational agility. Examples include Bahrami et al. [8], Kiani et al. [10], Pourkiani and Hojinipour [11], Sohrabi et al. [12], Bagherzadeh and Dibavar [13] Shiri et al. [14]. On the other hand, by examining the literature, it can be deduced that organizational intelligence can indirectly affect organizational agility through factors such as organizational forgetting and consequently organizational learning, which examples include Kiani et al. [10], Bahrami et al. [8] and [15].

Organizational forgetting is the consequence of a set of intra and extra organizational actions in which an organization loses some of its existing knowledge consciously (Purposeful forgetting) or unknowingly (Random forgetting). De Hallen and Phillips considered organizational forgetting based on two dimensions (targeted forgetting versus random forgetting) [16].

A hospital is an organization that has many interactions between nurses and patients, nurses and doctors, and doctors and patients, all of which can lead to learning and gaining experience [17]. Learning as one of the foundations of knowledge management deployment is one of the tools to gain competitive advantage for current organizations [18]. Sayert and March argue that learning is an effective strategy to increase organizational productivity [19]. Studies have been done on the level of health and treatment in nurses that have measured their learning capacity [17, 20, 21]. From Peter Senge's perspective, organizational learning consists of five components of individual skills, mental models, common vision, team learning and systemic thinking.

It is vital to understand the nature of change and the importance of future development for organizations, communities and even individuals planning for their future. The purpose of this study was to agility, intelligence, learning and forgetting modeling using structural equation method in general teaching hospitals of Shiraz University of medical sciences.

## Main text

### Materials and methods

**Fig. 1:**
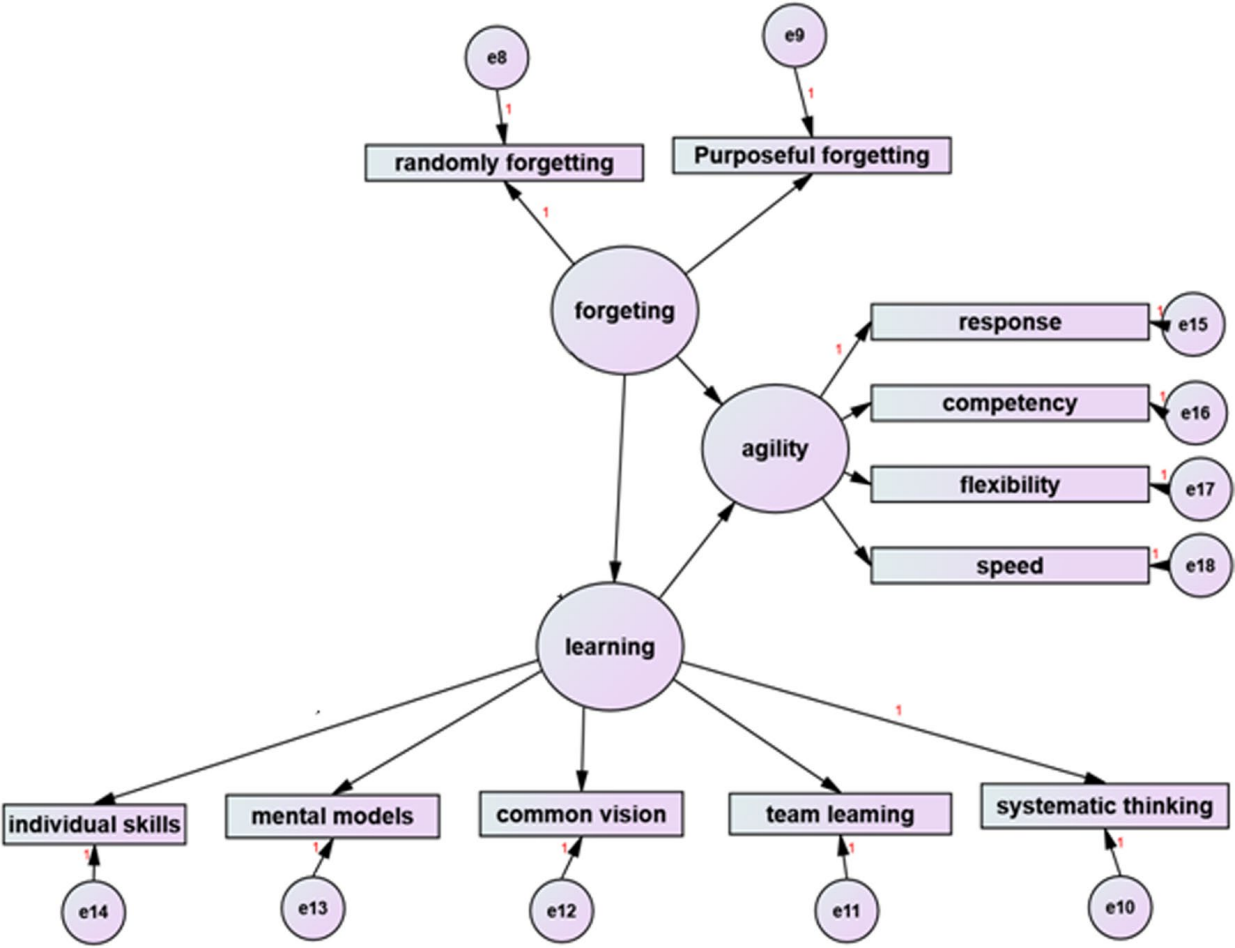
The hypothetical model derived from the theoretical literature of research

**Fig. 2:**
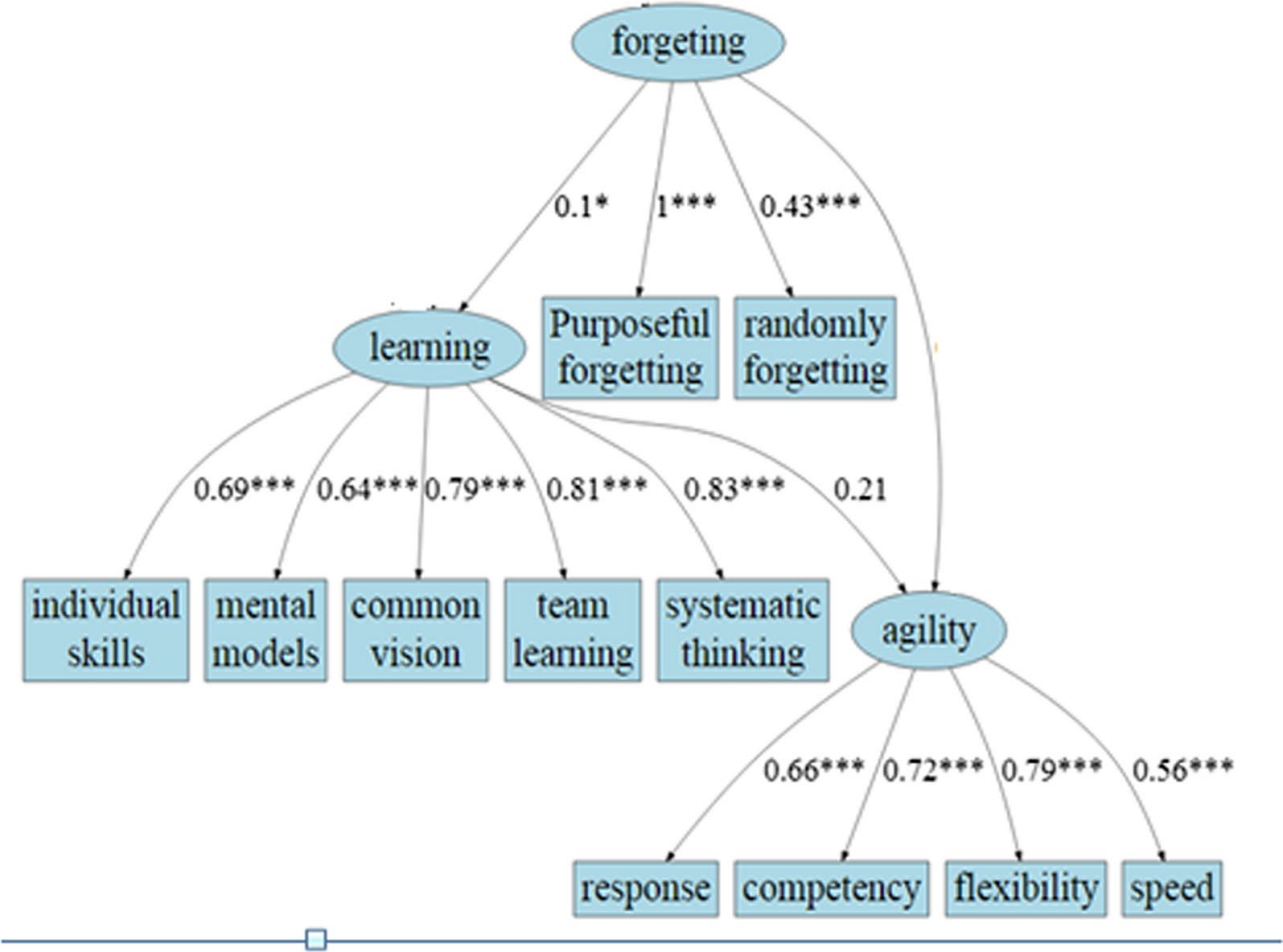
Research conceptual model and standardized indexes

This cross-sectional study was performed in the public-teaching hospitals (A and B) of Shiraz University of Medical Sciences in 1397. A hypothetical model was presented in order to investigate the relationship between the four variables of organizational intelligence, organizational learning, organizational forgetting and organizational agility. The first step in the structural equation modeling was to draw the causal graph of Fig. 1 (derived from theoretical literature) using AMOS software. Second step is sampling and experimental data collection and third step is using Structural Equation Method (SEM) and answer to the question whether the empirical data collected supports the theoretical model, OR NOT. The theoretical model of the study included a structural and a measurement model that were tested by the Structural Equation Method (SEM). The importance of this technique in health science research is that often in this field of study, researchers examine the relationships between different variables in the form of a model or network of relationships; therefore, based on their hypotheses about the relationships between variables, they design an overview of these relationships in the form of a prefabricated model. This method has been selected as a suitable technique to test the complex relationships between existing variables and also to overcome the limitations of traditional methods of analysis. The fifth step was to obtain the results of the theoretical model test (Fig. 1) and to reach the conceptual model (Fig. 2).

The statistical population consisted of 2000 administrative and medical staff. Sample size for Structural Equation Method estimated 342 people. Due to fall and not filling of questionnaires 10% of this sample size was not considered. Finally, the final sample size was 375 [22] and 316 questionnaires (84.2% out of 375 sample) were completed. The sampling method was stratified-random and the proportional allocation method. The criterion for the study was to have at least one year of work experience in the hospital.

The four standard questionnaires were used for data collection including: (1) The Persian version of Albert Sheet's (2003) organizational intelligence questionnaire [23], which was validated by Tabersa et al. (2012), and was used to collect data on components of organizational intelligence. (2) The Persian version of the Nifeh Organizational Learning Standard Questionnaire (2001), which was validated by Bidbakhti et al. (2014) and was used to collect data on organizational learning components [24]. (3) The Persian version of the standard organizational agility questionnaire based on Sharifi and Zhang's (1999) theory developed by Nikpour and Salajeghah (2010) that was used to collect data on the components of organizational agility [25]. (4) The questionnaire of organizational forgetting developed by Moshabbaki et al. with appropriate validity and reliability [26].

After data collection, questionnaires were entered into SPSS 21 software, then descriptive statistics indices such as frequency tables and standard deviation and appropriate charts were calculated and plotted. Analytical statistics were analyzed by R software. Structural equation modeling (SEM) method was used to analyze the data and evaluate the goals. To evaluate the effect of demographic variables, the total squares and Tukey tests were used.

### Results

Among these 316 samples, 240 people (75.9%) belonged to Hospital A and 76 people (24.1%) belonged to Hospital B, of which 26 people (8.2%) were administrative staff and 290 people (91.8%) Were medical staff. The majority of the sample consisted of 206 (65.2%) women.

The highest level of education was in the bachelor's degree (71.5%) and the lowest was in the diploma and lower's degree (2.8%); mean and standard. error of work experience was 8 ± 0/45 years and mean and standard. Error of The researchers age were 31/55 ±0/47.

According to Table 1, mean scores of organizational agility, intelligence, and learning were lower than average in both hospitals A and B.

**Table 1:** Descriptive statistics of latent variable scores for hospital A and B

		n	Learning		Forgetting		Smartness		Agility	
			Mean	Standard deviation	Mean	Standard deviation	Mean	Standard deviation	Mean	Standard deviation
Hospital	A	240	−4.495	2.994	.093	8.661	− 1.335	4.254	−.258	1.295
	B	76	−4.814	2.950	− .349	6.266	− 2.057	3.737	−.375	1.290

#### Research conceptual model test

Figure 2 shows the indices of conceptual model fitting; the tested model in this study had a higher score than all of the fitted indices that indicating good model quality.

Additional file 1: Table S1 shows the standardized path coefficients for the conceptual model. This image shows the relationships between the four variables of organizational intelligence, organizational learning, organizational forgetting and organizational agility as Latent variables, as well as the relationships of Latent variables with Observed variables with their coefficients of influence.

According to Additional file 2: Table S2, organizational intelligence had an indirect effect on organizational agility through organizational forgetting with path coefficient of 0.012. But through organizational learning no significant effect was observed. Organizational forgetting also had an indirect effect on organizational agility through organizational learning with path coefficient of 0.019.

According to Additional file 3: Table S3, which shows the relationship between Latent and Observed variables, there was relationship between the Latent variables and all of their Observed components. The latent variable of organizational intelligence is better represented by the Observed component of heart with the path coefficient of 1.172. The Latent variable of organizational learning is better represented by Observed component of mental models with path coefficient of 1.125. The Latent variable of organizational forgetting is also better represented through the Observed component of purposeful forgetting and finally the Latent variable of organizational agility can be better represented by the Observed component of competence. According to Additional 4: Table S4, which shows the direct and significant impact of Latent variables, organizational intelligence with path coefficient of 0.172 and organizational forgetting with path coefficient of 0.025 had a direct and significant effect on organizational agility (positive effect) but there was no direct and significant effect on organizational agility.

### Discussion

Organizational agility is one of the factors affecting the success of organizations in the competitive market; Study of factors affecting organizational agility in general teaching hospitals of Shiraz University of Medical Sciences. Based on the results of the analytical statistics related to the test of the conceptual model of the research with respect to the absolute fit index, Adaptive Fit Indexes and Model Economic Indicators and their comparison with their standard values is observed that theoretical model that is obtained using articles and references and suggested by the researcher consistent with the model obtained from empirical data and is a graceful model. Among the above indices, the most important index is CMIN, which is a very desirable value indicating the high quality of the conceptual model.

Based on the results of standard coefficients, regression and correlation coefficients and according to the fitted conceptual model, organizational agility score is below average in both Hospital A and Hospital B. The reason for the low average agility score in the two hospitals can be searched in Latent variables affecting the level of organizational agility. On the relation between Latent variables, there was a positive and significant relationship between organizational agility and organizational intelligence which is in line with the study of Bagherzadeh and Akbari Dibavar [27] and Purkiani et al. [11]. This means that the higher the average of organizational intelligence, the greater the agility of hospitals [28].

There was no significant positive relationship between agility and organizational learning. Ahmadi et al. in their study of the relationship between organizational agility and organizational learning in the Ministry of Sport and Youth showed that the Ministry of Sports and Youth Experts' perception of their organization as a learning organization and there is a significant correlation between organizational learning and organizational agility [29]. Hop and Ein found in their research that employees who go through different training courses can guarantee agility because these staff are flexible and can be moved wherever they need to. A workforce that has multiple skills performs better and has a lower error rate [30].

There was a positive and significant relationship between organizational agility and organizational forgetting. According to Sadeghian et al. study, there is a positive and meaningful relationship between purposeful organizational forgetting and organizational agility [37] and these results are in line with the findings of Zheng and Chen and Kizi and Oliviera [31, 32].On the other hand, in addition to organizational intelligence and organizational forgetting directly affect agility, can also influence agility indirectly and by reinforcing each other and through learning, so there was a positive and significant relationship between organizational intelligence and organizational learning. That means, for one unit of intelligence increase, which was matches with Hosseini's study [33].

There was a positive and significant relationship between organizational learning and organizational forgetting, which was not compatible with the study of Suri Zehi et al. Because these two variables were negatively and significantly correlated in the study by Suri Zehi et al. [32]. But it is in line with Jalali Farahani et al.'s study of explaining the relationship between purposeful organizational forgetting and organizational learning with organizational agility in Hamadan province's General Office of Sport and Youth which indicates a positive and meaningful relationship between purposeful organizational forgetting and organizational learning. It goes without saying that an organization often has to abandon its old knowledge in order to increase organizational knowledge and learning that impedes organizational learning [34].

Organizational forgetting as a mediating role between organizational intelligence and organizational learning had an indirect effect and has overall effect on agility. This is consistent with the study of Jalali et al. [34]. Consequently, it is very important to be aware of the Latent components of organizational intelligence, organizational learning, and organizational forgetting in the two hospitals A and B.

The mean scores of the Latent components results indicate the need for these hospitals to move towards becoming more intelligent and learner. In fact, management in these hospitals does not create a culture that considers the acquisition, creation and transfer of knowledge as a fundamental value in the organization. This finding is consistent with Yaghoubi et al.'s study of the relationship between components of organizational learning and knowledge management in selected hospital staffs in Isfahan, where the average organizational learning study was low [35]. The mean score of organizational forgetfulness in Hospital A is slightly above average and in Hospital B is below average. So if we want to have an agile hospital, we must first strengthen the levels of these three variables (organizational intelligence, organizational learning, and organizational forgetting) in two hospitals A and B. According to the conceptual model of research, we can see that there is a positive and significant relationship between the components of individual skills, mental models, common vision, team learning and systematic thinking with their Latent variable, namely organizational learning.

There was a significant positive relationship between the components of Purposeful forgetting and random forgetting with the Latent variable of organizational forgetting, It has match with the study by Haji Aziz et al. [36]. There was a positive and significant relationship between the Observed components of strategic approach, common file, tend to change, heart, alliance and agreement, Spreading of knowledge and performance pressure with the latent variable of organizational intelligence, which consistent with the study by Hosseini et al. [33]. There was a positive correlation between the observed components of response, competency, flexibility and speed with the Latent variable of organizational agility. It was consist with Gunasekaran et al. [37].

## Conclusion

Service organizations, including hospitals, are increasingly in need of agility because it not only achieves their goals but also concerns the lives of people, doubling their responsibilities. By correctly identifying organizational learning, organizational intelligence and organizational forgetting, organizations can be placed in the right position in terms of agility. Therefore, as organizational intelligence, organizational forgetting and organizational learning increase, it has a greater impact on organizational agility and enhances organizational agility, which in turn will increase the effectiveness and efficiency of activities in the organization. For agility, hospitals need to focus on enhancing the Observed components of each of these Latent variables. These components include strategic approach, common file, tend to change, heart, alliance, agreement, spreading of knowledge and performance pressure to enhance organizational intelligence; personal skills, mental models, common vision, team learning and systematic thinking to enhance organizational learning and Purposeful forgetting and random forgetting to enhance organizational forgetting are noted. Among these components, organizational intelligence with more heart, organizational learning with mental models, and organizational forgetting with purposeful organizational forgetting are finding outcome. As a result, if hospital managers seek to perceive change in these three Latent variables (intelligence, learning, and forgetting) and then organizational agility, they should focus on these three components (heart, mental models, and Purposeful organizational forgetting).

Ultimately, the most important factor in increasing the agility of organizations is to pay attention to the components that, if they are specifically targeted, organizations will achieve agility directly. These factors include the ability to detect and respond to changes quickly and efficiently, the ability to achieve organizational goals and objectives, the ability to streamline different processes and achieve different goals using the same facilities and capabilities, and the ability to perform activities in the least possible time.

## Limitations

One of the limitations of this study is the lack of information of the administrative-medical staff of hospitals regarding the subject of research. The study sought to address some ethical considerations, such as: giving staff sufficient information about the research and allowing them to participate in the research, being totally neutral and avoiding any explanation that could lead to a specific response, complete accuracy of data collection and analysis, non-distortion of results, confidentiality.

## Supplementary Information

**Additional file 1: Table S1.** Results indicators conceptual model.

**Additional file 2: Table S2.** Effects of research variables, standardized coefficients, and standardized solution (fit).

**Additional file 3: Table S3.** Standardized estimations and their standard errors for parameters corresponding to Fig. 1.

**Additional file 4: Table S4.** Standardized estimations and their standard errors for parameters corresponding to Fig. 1(latentvariable).

## Data Availability

The present research data will be available upon logical request. If anyone wants to request data, they should contact MS.

## Abbreviations

AGFI: Adjusted goodness of fit
CFI: Comparative fit index
Df: Difference
GFI: Goodness of fit
NFI: Normed fit index
NNFI: Nonnormed fit index
PNEI: Parsimonious normed fit index
RFI: Relative fit index
RMSEA: Root mean square error of approximation
