# Supplementary material for: Modeling organizational intelligence, learning, forgetting and agility using structural equation model approaches in Shiraz University of Medical Sciences Hospitals
Source: BMC Res Notes. 2021 Jul 21;14:277. doi: 10.1186/s13104-021-05682-w (PMC8293499; doi:10.1186/s13104-021-05682-w)
Supplement: Supplementary file 1 — Additional file 1: Table S1. Results indicators conceptual model. [file 13104_2021_5682_MOESM1_ESM.docx]

Table S1 . Results indicators conceptual model.

| **Acceptable value** | **Index value** | **Symbol** |
| --- | --- | --- |
| **>90** | 0.936 | GFI |
| **>90** | 0.913 | AGFI |
| **>90** | 0.963 | NNFI |
| **>90** | 0.919 | NFI |
| **>90** | 0.970 | CFI |
| **>90** | 0.901 | RFI |
| **>90** | 0.970 | IFI |
| **>0/5** | 0/757 | PNFI |
| **<0/05** | 0/041 | RMSEA |
| **<3** | 1/539 | CMIN |

Data are presented as % unless otherwise indicated. AGFIZadjusted

goodness of fit; CFIZcomparative fit index; dfZdifference;

GFIZgoodness of fit; IFIZ; NFIZnormed fit index; NNFIZnonnormed

fit index; PNEI Z parsimonious normed fit index;

RFIZrelative fit index; RMSEAZroot mean square error of

approximation.
