# Supplementary material for: Modeling organizational intelligence, learning, forgetting and agility using structural equation model approaches in Shiraz University of Medical Sciences Hospitals
Source: BMC Res Notes. 2021 Jul 21;14:277. doi: 10.1186/s13104-021-05682-w (PMC8293499; doi:10.1186/s13104-021-05682-w)
Supplement: Supplementary file 2 — Additional file 2: Table S2. Effects of research variables, standardized coefficients, and standardized solution (fit). [file 13104_2021_5682_MOESM2_ESM.docx]

Table S2. Effects of research variables, standardized coefficients, and standardized solution (fit).

| Indirect effect | Total effect | Variable |
| --- | --- | --- |
| - | 0/226 | Organizational intelligence , organizational agility th Organizational learning |
| 0/012 | 0/184 | Organizational intelligence , organizational agility th Organizational forgeting |
| 0/019 | 0/572 | Organizational intelligence , Organizational learning th organizational forgeting |
| 0/019 | 0/572 | Organizational forgetting, organizational agility th organizational learning |

*Th: through*
