# Supplementary material for: Modeling organizational intelligence, learning, forgetting and agility using structural equation model approaches in Shiraz University of Medical Sciences Hospitals
Source: BMC Res Notes. 2021 Jul 21;14:277. doi: 10.1186/s13104-021-05682-w (PMC8293499; doi:10.1186/s13104-021-05682-w)
Supplement: Supplementary file 3 — Additional file 3: Table S3. Standardized estimations and their standard errors for parameters corresponding to Fig. 1. [file 13104_2021_5682_MOESM3_ESM.docx]

1Table S3: standardized estimations and their standard errors for parameters corresponding to figuer 1.

| P(>\|z\|) | z-value | Std.Err | Estimate |  | row |
| --- | --- | --- | --- | --- | --- |
| - | - | - | 1.000 | Intelligence =~ strategic approach | 1 |
| 0/000 | 4/108 | 0/210 | 0/862 | Intelligence =~com fate | 2 |
| 0/000 | 9/001 | 0/092 | 0/824 | Intelligence =~tendency to change | 3 |
| 0/000 | 9/182 | 0/128 | 1/172 | Intelligence =~heart | 4 |
| 0/000 | 8/518 | 0/126 | 1/074 | Intelligence=~ alignment | 5 |
| 0/000 | 8/794 | 0/119 | 1/046 | Intelligence =~ expanding knowledge | 6 |
| 0/000 | 3/694 | 0/282 | 1/041 | Intelligence =~ pressure of operation | 7 |
| 0/000 | - | - | 1/000 | Learning =~ individual skills | 8 |
| 0/000 | 10/217 | 0/110 | 1/125 | Learning =~ mental model | 9 |
| 0/000 | 12/676 | 0/063 | 0/795 | Learning =~ com vision | 10 |
| 0/000 | 12/740 | 0/062 | 0/790 | Learning =~ team learning | 11 |
| 0/000 | 12/448 | 0/070 | 0/872 | Learning =~ systems thinking | 12 |
| 0/000 | - | - | 1/000 | Forgetting =~ purposed | 13 |
| 0/000 | 8/347 | 0/050 | 0/421 | Forgetting =~ randomly | 14 |
| 0/000 | - | - | 1/000 | Agility =~ respond | 15 |
| 0/000 | 12/269 | 0/201 | 2/471 | Agility =~ the competency | 16 |
| 0/000 | 10/616 | 0/116 | 1/226 | Agility =~ flexibility | 17 |
| 0/000 | 8/395 | 0/157 | 1/316 | Agility =~ speed | 18 |

*=~: shows path for covariance and so on*
