# Supplementary material for: Modeling organizational intelligence, learning, forgetting and agility using structural equation model approaches in Shiraz University of Medical Sciences Hospitals
Source: BMC Res Notes. 2021 Jul 21;14:277. doi: 10.1186/s13104-021-05682-w (PMC8293499; doi:10.1186/s13104-021-05682-w)
Supplement: Supplementary file 4 — Additional file 4: Table S4. Standardized estimations and their standard errors for parameters corresponding to Fig. 1(latentvariable). [file 13104_2021_5682_MOESM4_ESM.docx]

Table S4: standardized estimations and their standard errors for parameters corresponding to figuer 1(latentvariable).

| P(>\|z\|) | z-value | Std.Err | Estimate |  | row |
| --- | --- | --- | --- | --- | --- |
| 0/000 | 4/290 | 0/040 | 0/172 | Intelligence ~Agility | 1 |
| 0/000 | 7/779 | 0/071 | 0/553 | Intelligence~ Learning | 2 |
| 0/050 | 1/959 | 0/050 | 0/097 | Learning~Agility | 3 |
| 0/000 | 4/042 | 0/119 | 0/482 | Intelligence~ forgetting | 4 |
| 0/006 | 2/773 | 0/009 | 0/025 | forgetting~Agility | 5 |
| 0/020 | 2/320 | 0/017 | 0/040 | Learning~ forgetting | 6 |
